# A cohort study of the occurrence of post-term births and its association with perinatal mortality in a rural area in Bangladesh

**DOI:** 10.7189/jogh.14.04238

**Published:** 2024-11-15

**Authors:** U Tin Nu, Jesmin Pervin, Monjur Rahman, Kazi Tamara B Kamal, Shaki Aktar, Fauzia A Huda, Shikha Ganguly, Shams El Arifeen, Lars Åke Persson, Anisur Rahman

**Affiliations:** 1 Maternal and Child Health Division, International Centre for Diarrhoeal Disease Research, Bangladesh (icddr,b), Dhaka, Bangladesh; 2 Department of Gynaecology and Obstetrics, Dhaka Medical College and Hospital, Dhaka, Bangladesh; 3 Department of Disease Control, Faculty of Infectious and Tropical Diseases, London School of Hygiene and Tropical Medicine, London, UK

## Abstract

**Background:**

We aimed to evaluate the trend of post-term births over time and their association with perinatal mortality based on prospective pregnancy cohorts in a rural area in Bangladesh.

**Methods:**

This cohort study included 72 373 singleton births with gestational ages ≥28 weeks recorded by a health and demographic surveillance system from 1990 to 2019 in Matlab, Bangladesh. We expressed the gestational age as X (weeks) + Y (days)/7 weeks, where X indicated complete weeks, and Y presented the number of completed days out of seven days or a week. Using Poisson regression with robust variances, we estimated the population-based proportion of post-term births and assessed the association between gestational age categories and perinatal mortality. We presented results by adjusted relative risk (aRR) with 95% confidence intervals (CIs).

**Results:**

Post-term births declined from 5.8% in 1990 to 2.8% in 2019. Perinatal mortality declined from 58 to 27 per 1000 births from 1990 to 2019. Compared to full-term births (39 + 0/7 to 40 + 6/7 weeks), the aRRs of perinatal mortality were 1.39 for late-term (41 + 0/7 to 41 + 6/7 weeks) and 1.93 for post-term (≥42 + 0/7 weeks) births. The population-attributable fraction of perinatal mortality was 15% for births at ≥41 + 0/7 weeks out of the total perinatal deaths occurring at ≥39 gestation weeks.

**Conclusions:**

In this rural setting in Bangladesh, we observed a decline in post-term birth proportions from 1990 to 2019. We found increased perinatal mortality when pregnancy continued beyond 40 + 6/7 weeks of gestation. This implies that planning the management of pregnant women approaching the post-term period may be needed to further improve perinatal health outcomes.

Post-term birth is birth at a gestation of ≥42 weeks [1–3]. It has been associated with increased perinatal morbidity and mortality [4–7]. The estimated global prevalence of post-term births ranges from 1–10% of all deliveries [8–10]. The occurrence differs within and between countries [11], partly depending on assessments by reported last menstrual period (LMP) dates or ultrasound scans in early pregnancy [11,12]. Local obstetric practices of labour induction and elective caesarean sections also influence the proportion of post-term births in a population [12–14].

Even in recent publications, the precise biological mechanisms of labour initiation and facilitation of its progression are unclear [12], although some hormonal and genetic factors are suggested. The placenta produces more corticotrophin-releasing hormones in late pregnancy, reaching a peak during labour and, therefore, is linked to the gestation length. The slow increase in corticotrophin-releasing hormone production has been associated with delayed onset of labour and prolonged gestation [12,15]. Several factors have been claimed to be associated with post-term birth, such as genetic predisposition [11,16], first-time pregnancy [1,16,17], older maternal age [16,17], lower maternal education [17], obesity [1,11], previous post-term birth [1,18], and male foetal sex [12,18]. Prolonged pregnancies contribute to severe morbidities for foetus and neonates that include placental insufficiency [19], labour dystocia [1,12], meconium aspiration [1,16,20], macrosomia [16], and birth asphyxia [11,18,21,22]. All these conditions have implications for foetal and neonatal survival [1,12,16].

A recent systematic review reported that the risk of stillbirth gradually increased from 0.11 to 3.00 per 1000 pregnancies from 37–42 weeks of gestation, and the risk of neonatal mortality increased two times for pregnancies at 42 weeks compared to 41 weeks of gestation [5]. However, all studies included in this review were conducted in high-income countries. Despite the importance of prolonged pregnancy continuing beyond 41 + 6/7 weeks (41 weeks and 6 days) for adverse health outcomes, clinical management per World Health Organization (WHO) guidelines is not consistently followed in most health facilities in low- and lower-middle-income countries, including Bangladesh [23].

In Bangladesh, the 2017–18 Demographic and Health Survey reported perinatal death (stillbirths and early neonatal deaths within the first seven days of life) and neonatal mortality rates at 48 per 1000 births and 30 per 1000 live births, respectively [24]. Furthermore, it is worth noting that eight out of 10 neonatal deaths occurred within seven days of life [24]. These rates are far away from the Every Newborn Action Plan and Sustainable Development Goals 2030 targets to reduce stillbirth and neonatal deaths to or below 12 per 1000 births and 12 per 1000 live births, respectively [25,26].

Earlier studies on prolonged pregnancy and perinatal outcomes were mainly conducted in high-income countries. The limited number of studies available from low-income settings have been based on hospital data [27–30]. Therefore, studies of the population-based occurrence of post-term birth and its association with perinatal mortality by gestational week are needed in low- and lower-middle-income country settings, including Bangladesh. These studies will help uncover the data gaps of the burden of post-term birth at the population level and the risk of perinatal deaths by each week of gestation so appropriate measures may be adopted by practitioners and policymakers to further improve perinatal health. In the present study, we aimed to estimate the population-based trend of post-term births and assessed the risks of perinatal mortality by gestational weeks at birth based on prospective pregnancy cohorts in the Health and Demographic Surveillance System (HDSS) in Matlab, Bangladesh, spanning from 1990 to 2019. We also calculated the population-attributable fraction (PAF) of perinatal mortality by birth at 41 + 0/7 gestation weeks and beyond.

## METHODS

### Study setting, design, and population

This cohort study was conducted in the HDSS of the International Centre for Diarrhoeal Disease Research, Bangladesh (icddr,b). The study site is in the Chandpur district’s Matlab North and South subdistricts. The HDSS includes continuous longitudinal recordings of demographic events of marriages, births, deaths, migrations, and divorces. In addition, the HDSS collects health events and selects reproductive health-related information such as pregnancy information. The Matlab HDSS employs a network of local female community health workers (CHWs) for data collection. The CHWs were scheduled to visit each household every fortnight up to 2001, monthly from 2002 to 2007, and thereafter with two-month intervals [31,32]. The CHWs used simple paper-based structured questionnaires for data collection since the initiation of HDSS. In 2010, they used personal digital assistants, followed by Android tablets in 2014 [31]. In this cohort study, we used all pregnancy data collected by CHWs from 1990 to 2019 by the HDSS.

### Data collection

Female CHWs visited married women of reproductive age in the surveillance population of about 230 000 during routine house-to-house visits [31]. The CHWs identified pregnant women by inquiring about the history of missed menstruation since their last household visit. To improve the pregnancy identification process and tracking of pregnant women, the icddr,b introduced urine pregnancy tests in 2001 and continued until 2004 as a part of a nutrition intervention trial in the study area [33]. This initiative was adopted as routine pregnancy surveillance in 2007 [34]. CHWs registered a woman as pregnant if she had missed menstruation longer than two weeks or tested positive by a urine pregnancy test. After identifying pregnant women, the CHWs recorded their LMP and followed up for pregnancy outcomes and dates.

The CHWs received adequate training on data collection on selected maternal and child events. Two tiers of supervision and an independent data management team have secured data quality and completeness. Field supervisors also perform random in-person checks of household visits to ensure data quality [35].

### Exposure

Maternal gestational age was considered the ‘exposure’ in the analysis and defined as the duration from the start of the first day of the LMP to the date of birth. We expressed the gestational age in X (weeks) + Y (days)/7 weeks, where X indicated complete weeks, and Y presented the number of completed days out of seven days or a week. Births from 37 + 0/7 to 41 + 6/7 weeks were considered term births based on gestational age. Births from 42 + 0/7 to 44 + 6/7 weeks were considered post-term births. The term births were further divided into early-term (37 + 0/7 to 38 + 6/7 weeks), full-term (39 + 0/7 to 40 + 6/7 weeks), and late-term (41 + 0/7 to 41 + 6/7 weeks) births [36]. The birth of any live-born baby before 37 completed weeks of gestation was considered pre-term birth [32]. To determine the PAF of perinatal death, the gestational week of birth was grouped into 39 + 0/7 to 40 + 6/7 weeks (full-term) and ≥41 + 0/7 weeks (late- and post-term).

### Outcome

The outcome of the present study was perinatal mortality, defined as the number of stillbirths and early neonatal mortality per 1000 births. Stillbirth was defined as the death of a foetus at or after 28 weeks of gestation. Early neonatal mortality was considered a newborn’s death within seven days after birth [34].

### Covariates

We identified the variables after reviewing the existing literature. Finally, we selected the covariates based on their availability in the HDSS databases. We obtained covariate information such as women’s age, education, household socioeconomic status, and birth year from the HDSS databases. Women were divided into four age groups (<20, 20–24, 25–29, and ≥30 years). Maternal education was categorised into four groups based on the completed number of years at school: no education (zero years), primary education (one to five years), secondary education (six to 10 years), and higher education (≥11 years). Parity was defined as the number of preceding births, including stillbirths, and grouped into 0, 1, and ≥2. We first generated wealth scores based on durable household assets, land possessions, and dwelling structures using principal component analysis to determine socioeconomic status. The computed wealth scores were then categorised into quintiles, where the poorest was represented by 1, and the wealthiest was 5 [37]. Birth years were grouped into three cohort periods: 1990–99, 2000–09, and 2010–19.

### Data analysis

The characteristics of study participants were presented in frequencies, proportions, and means and standard deviation (SD). We graphically presented the distribution of birth categories by gestational age and perinatal mortality over the study period. We used the χ^2^ test to assess the associations between available covariates and perinatal mortality. We examined the association between gestational age categories (pre-term, early-term, full-term, late-term, and post-term) and perinatal mortality, fitting log-linear Poisson regression models with robust variance. Poisson regressions were considered an appropriate method to analyse rare events when the subjects were followed up for a long period. We employed Poisson regression models with robust variance after encountering non-convergence issues while attempting to fit the log-binomial regression model [38]. We also performed Deviance and Pearson goodness-of-fit tests. We observed that the model was not statistically significant, indicating that it fits the data well. A potential confounder was included for adjustment if it was associated with perinatal mortality at a *P*-value <0.05 level in the final model. The results were presented as adjusted relative risks (aRRs) with 95% confidence intervals (CIs). We also assessed the risks by three 10-year cohort periods (1990–99, 2000–09, and 2010–19) to judge the robustness of associations. Finally, we also determined the PAF of perinatal mortality due to prolonged pregnancy out of the total births at ≥39 + 0/7 gestational weeks based on the formula, as suggested by Levin [39,40]:



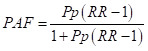



Here, *Pp* indicates the prevalence of exposures (gestational weeks of births at ≥41 + 0/7 weeks) in the population, and *RR* represents the relative risk of perinatal mortality in the exposed group (≥41 + 0/7 weeks) compared to the unexposed (39 + 0/7 to 40 + 6/7 weeks) group. The data were analysed using Stata, version 16 (StataCorp, College Station, Texas, USA).

## RESULTS

**Figure 1:**
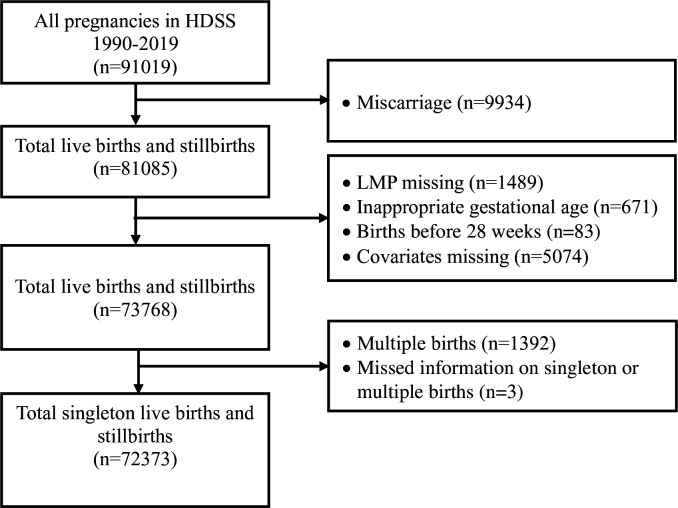
Study flowchart.

The HDSS databases contained information on 91 019 pregnancies from 1 January 1990 to 31 December 2019. After excluding early miscarriages (n = 9934), we had 81 085 births available. Of these total births, 72 373 singleton births from 28 + 0/7 to 44 + 6/7 weeks of gestation were available after excluding improbable or missing LMP, missing covariates, and multiple births to estimate the population-based post-term birth proportions (**Figure 1**).

### Maternal characteristics

Women’s mean age was 25.4 years (SD = 5.8), and one-third of the women were aged 20–24 years. About two-fifths of the women were nulliparous, and four-tenth of the women had completed secondary (six to 10 years) education. Approximately three-tenth underwent caesarean sections (**Table 1**).

**Table 1:** Cohort and birth characteristics of all women by gestational age groups by year from 1990 to 2019 in Matlab, Bangladesh*

	Gestational age in weeks at birth (n = 72 373)						
**Characteristics**	**Total birth (28–44)**	**Pre-term (<37)**	**Early-term (37–38)**	**Full-term (39–40)**	**Late-term (41)**	**Post-term (42–44)**	***P*-value**†
Total	72 373 (100.0)	13 033 (18.0)	19 778 (27.3)	28 632 (39.6)	6853 (9.5)	4077 (5.6)	
Maternal age in years							<0.001
*<20*	14 031 (19.4)	2426 (18.6)	3387 (17.1)	5890 (20.6)	1488(21.7)	840 (20.6)	
*20–24*	23 618 (32.6)	4005 (30.7)	6176 (31.2)	9675 (33.8)	2430 (35.5)	1332 (32.7)	
*25–29*	18 654 (25.8)	3153 (24.2)	5384 (27.2)	7289 (25.5)	1729 (25.2)	1099 (27.0)	
*≥30*	16 070 (22.2)	3449 (26.5)	4831 (24.4)	5778 (20.2)	1206 (17.6)	806 (20.0)	
Education in years							<0.001
*0*	19 967 (27.6)	4993 (38.3)	5496 (27.8)	6560 (22.9)	1647 (24.0)	1271 (31.2)	
*1–5*	18 020 (24.9)	3633 (27.9)	4937 (25.0)	6735 (23.5)	1617 (23.6)	1098 (26.9)	
*6–10*	31 114 (43.0)	4089 (31.4)	8380 (42.4)	13 806 (48.2)	3245 (47.4)	1594 (39.1)	
*≥11*	3272 (4.5)	318 (2.4)	965 (4.9)	1531 (5.4)	344 (5.0)	114 (2.8)	
Wealth quintiles							<0.001
*1-poorest*	12 981 (17.9)	2656 (20.4)	3505 (17.7)	4827 (16.9)	1195 (17.4)	798 (19.6)	
*2*	12 724 (17.6)	2461 (18.9)	3560 (18.0)	4827 (16.9)	1175 (17.1)	701 (17.2)	
*3*	14 283 (19.7)	2594 (19.9)	3875 (19.6)	5655 (19.8)	1346 (19.6)	813 (19.9)	
*4*	15 666 (21.6)	2709 (20.8)	4276 (21.6)	6264 (21.9)	1500 (21.9)	917 (22.5)	
*5-wealthiest*	16 719 (23.1)	2613 (20)	4562 (23.1)	7059 (24.7)	1637 (23.9)	848 (20.8)	
Parity							<0.001
*0*	26 988 (37.3)	4467 (34.3)	6457 (32.6)	11 517 (40.2)	2997 (43.7)	1550 (38.0)	
*1*	20 534 (28.4)	3323 (25.5)	6134 (31.0)	8154 (28.5)	1802 (26.3)	1121 (27.5)	
*≥2*	24 851 (34.3)	5243 (40.2)	7187 (36.3)	8961 (31.3)	2054 (30.0)	1406 (34.5)	
Pregnancy outcome							<0.001
*Stillbirth*	1559 (2.2)	631 (4.8)	308 (1.6)	381 (1.3)	131 (1.9)	108 (2.6)	
*Livebirth*	70 814 (97.8)	12 402 (95.2)	19 470 (98.4)	28 251 (98.7)	6722 (98.1)	3969 (97.4)	
Perinatal death							<0.001
*No*	69 584 (96.1)	11 830 (90.8)	19 265 (97.4)	27 981 (97.7)	6626 (96.7)	3882 (95.2)	
*Yes*	2789 (3.9)	1203 (9.2)	513 (2.6)	651 (2.3)	227 (3.3)	195 (4.8)	
Delivery type‡							<0.001
*Vaginal delivery*	25 134 (69.6)	3076 (69.8)	6670 (65.4)	11 543 (71.7)	2591 (69.5)	1254 (74.4)	
*Caesarean section*	10 988 (30.4)	1328 (30.2)	3528 (34.6)	4561 (28.3)	1139 (30.5)	432 (25.6)	
Birth year							<0.001
*1990–99*	23 793 (32.9)	6230 (47.8)	6345 (32.1)	7815 (27.3)	1920 (28)	1483 (36.4)	
*2000–09*	24 478 (33.8)	4063 (31.2)	6333 (32.0)	9617 (33.6)	2675 (39)	1790 (43.9)	
*2010–19*	24 102 (33.3)	2740 (21.0)	7100 (35.9)	11 200 (39.1)	2258 (32.9)	804 (19.7)	

*Presented as n (%) unless specified otherwise.

†Based on χ^2^ tests between gestational age groups.

‡Available from 2005.

### Trends in post-term births

**Figure 2:**
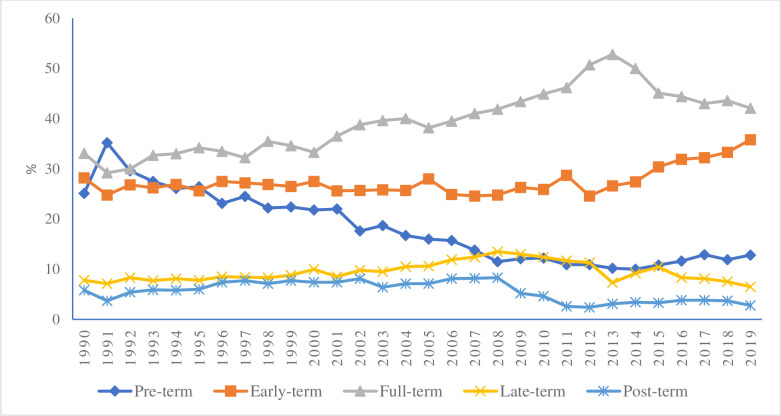
Distribution of pre-term (<37 weeks), early-term (37 + 0/7 to 38 + 6/7 weeks), full-term (39 + 0/7 to 40 + 6/7 weeks), late-term (41 + 0/7 to 41 + 6/7 weeks), and post-term (42 + 0/7 to 44 + 6/7 weeks) births by year from 1990 to 2019 in Matlab, Bangladesh.

The average proportion of post-term births among all singleton births, from 28 + 0/7 to 44 + 6/7 weeks of gestation, was 5.6% across the study period. The post-term birth proportions declined from 5.8% in 1990 to 2.8% in 2019. From 1996 to 2007, the proportions remained relatively stable, ranging between 7.4% and 8.3%, but exhibited a sharp decline after that. The proportion of full-term births increased from 38.2% in 2005 to 52.8% in 2013, then declined to 42.1% in 2019. We also observed a similar increased pattern of early-term birth proportions (**Figure 2**). We observed a substantial rise in caesarean sections from 7% to 56.4% between 2005–19 (Figure S1 in the **Online Supplementary Document**). The increase in early-term and full-term births and decrease in post-term birth proportions corresponded to a high proportion of caesarean section deliveries from 2008 (**Figure 2**, Figure S1 in the **Online Supplementary Document**). The average proportion of pre-term births among all singleton births was 18%. We observed a substantial decline in pre-term births from 25.1% to 12.8% between 1990–2019, and these results have already been published [32,41].

### Perinatal mortality

**Figure 3:**
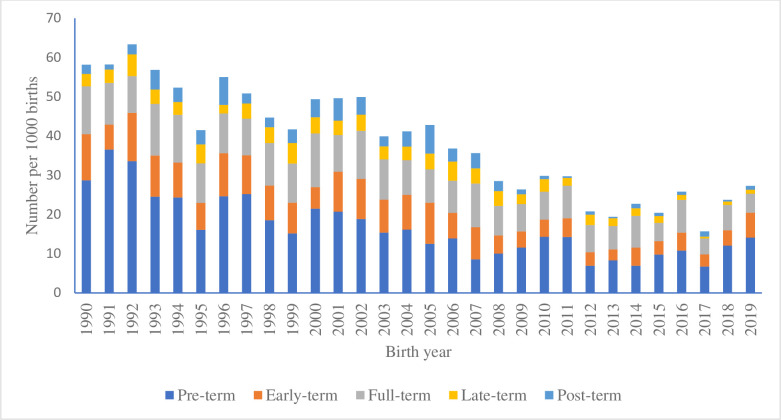
Distribution of perinatal death by pre-term (<37 weeks), early-term (37 + 0/7 to 38 + 6/7 weeks), full-term (39 + 0/7 to 40 + 6/7 weeks), late-term (41 + 0/7 to 41 + 6/7 weeks), and post-term (42 + 0/7 to 44 + 6/7 weeks) births by year from 1990 to 2019 in Matlab, Bangladesh.

Of 2789 perinatal deaths among births at ≥28 + 0/7 gestational weeks, 1559 were stillbirths, and the remaining were early neonatal deaths (**Table 1**). The proportion of perinatal mortality was reduced from 58 per 1000 births in 1990 to 27 per 1000 births in 2019 (**Figure 3**). During the same period, stillbirths were reduced from 30.7 to 12.7 per 1000 births, and early neonatal mortality from 28.4 to 14.8 per 1000 live births. The results indicate a substantial decrease in perinatal mortality in the study area.

**Figure 4:**
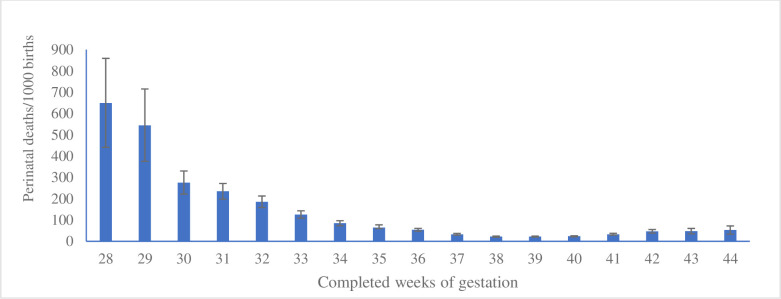
Distribution of perinatal deaths by gestational weeks at birth in the pregnancy cohorts by year from 1990 to 2019 in Matlab, Bangladesh.

Among births at a given gestational week, the proportion of perinatal mortality was 650 per 1000 births at 28 weeks. It then sharply decreased to 54 per 1000 births at 36 weeks. At week 37, the mortality was 33 per 1000 births, decreased further at 38 weeks, and remained similar up to 40 weeks of births. The perinatal mortality then started increasing from 41 + 0/7 weeks to continue up to 44 + 6/7 weeks of gestation (late-term and post-term births) at births (**Figure 4**).

### Association of covariates and gestational age with perinatal mortality

Among the available covariates, maternal age, parity, education, and birth year were associated (*P* < 0.05) with perinatal mortality (**Table 2**). In the multivariable model, maternal age, parity, education, and birth year were associated with perinatal mortality. The risks of perinatal mortality were 1.7 and about 2.2 times higher in the no education and primiparity groups compared to the group with higher education (≥11 years) and high parity groups (≥2), respectively (Table S1 in the **Online Supplementary Document**).

**Table 2:** Association of perinatal death with available covariates in the pregnancy cohorts by year from 1990 to 2019 in Matlab, Bangladesh (n = 72 373), presented as n (%)

	Perinatal death		
**Characteristics**	**No**	**Yes**	***P*-value**†
Maternal age in years			<0.001
*<20*	13 446 (19.3)	585 (21.0)	
*20–24*	22 748 (32.7)	870 (31.2)	
*25–29*	18 033 (25.9)	621 (22.3)	
*≥30*	15 357 (22.1)	713 (25.6)	
Education in years			<0.001
*0*	18 897 (27.2)	1070 (38.4)	
*1–5*	17 333 (24.9)	687 (24.6)	
*6–10*	30 160 (43.3)	954 (34.2)	
*≥11*	3194 (4.6)	78 (2.8)	
Wealth quintiles			0.119
*1-poorest*	12 453 (17.9)	528 (18.9)	
*2*	12 246 (17.6)	478 (17.1)	
*3*	13 708 (19.7)	575 (20.6)	
*4*	15 052 (21.6)	614 (22.0)	
*5-wealthiest*	16 125 (23.2)	594 (21.3)	
Parity			<0.001
*0*	25 432 (36.5)	1556 (55.8)	
*1*	20 014 (28.8)	520 (18.6)	
*≥2*	24 138 (34.7)	713 (25.6)	
Birth year			<0.001
*1990–99*	22551 (32.4)	1242 (44.5)	
*2000–09*	23499 (33.8)	979 (35.1)	
*2010–19*	23534 (33.8)	568 (20.4)	

*Presented as n (%) unless specified otherwise.

†Based on χ^2^ tests.

The aRR of perinatal death was 1.93 (95% CI = 1.65–2.25) for post-term (42 + 0/7 to 44 + 6/7 weeks) births and 1.39 (95% CI = 1.20–1.61) for late-term (41 + 0/7 to 41 + 6/7 weeks), respectively, compared to the full-term (39 + 0/7 to 40 + 6/7 weeks) births. Deliveries at early-term (37 + 0/7 to 38 + 6/7 weeks) had 1.15 times (95% CI = 1.03–1.29) higher risk of perinatal death compared to full-term births (39 + 0/7 to 40 + 6/7 weeks). Pre-term births (<37 weeks) had a 3.61 (95% CI = 3.28–3.98) times greater risk of perinatal death than full-term births (**Table 3**). The above results and the perinatal death risks analysed for each week of gestation revealed that birth at a full-term gestation period had the safest window for delivery (**Table 3**, Table S2 in the **Online Supplementary Document**).

**Table 3:** Association of gestation age categories with perinatal death in the pregnancy cohorts by year from 1990 to 2019 in Matlab, Bangladesh (n = 72 373)

Gestation at delivery in weeks	Births (n)	Perinatal deaths, n (%)	RR (95% CI)	*P*-value	aRR (95% CI)*	*P*-value
Pre-term (<37)	13 033	1203 (9.2)	4.06 (3.70–4.46)	<0.001	3.61 (3.28–3.98)	<0.001
Early-term (37–38)	19 778	513 (2.6)	1.14 (1.02–1.28)	0.024	1.15 (1.03–1.29)	0.015
Full-term (39–40)	28 632	651 (2.3)	Ref.		Ref.	
Late-term (41)	6853	227 (3.3)	1.46 (1.26–1.70)	<0.001	1.39 (1.20–1.61)	<0.001
Post-term (42–44)	4077	195 (4.8)	2.10 (1.80–2.46)	<0.001	1.93 (1.65–2.25)	<0.001

aRR – adjusted relative risks, CI – confidence interval, ref – reference, RR – relative risks

*Adjusted for maternal age, education, parity, and birth year.

We observed similar risk estimates of perinatal morality for post-term birth throughout the three 10-year study periods. Between delivery types (vaginal or caesarean section), we also observed similar risk estimates of perinatal mortality (Tables S3–4 in **the** **Online Supplementary Document**).

We estimated that 15% of the perinatal mortality could be avoided at ≥41 + 0/7 gestational weeks if the labour was initiated between 39 + 0/7 and 40 + 6/7 gestational weeks (PAF = 15, 95% CI = 14.3–15.7) (Table S5 in the **Online Supplementary Document**).

## DISCUSSION

In this population-based pregnancy cohort over 30 years in a rural area in Bangladesh, we observed that the proportion of post-term births was reduced from 5.8% in 1990 to 2.8% in 2019, with an average of 5.6%. Across the same period, the perinatal mortality declined from 58 to 27 per 1000 births. Perinatal mortality was about two times higher for post-term births (42 + 0/7 to 44 + 6/7 weeks) than for full-term births (39 + 0/7 to 40 + 6/7 weeks). The risks of perinatal mortality started to increase even from late-term (41 + 0/7 to 41 + 6/7 weeks) births compared to births at full-term. Furthermore, about one in seven perinatal deaths could be attributed to births beyond 40 + 6/7 weeks of gestation.

Population-based studies addressing trends in post-term births in low- and lower-middle-income countries are scarce. Previous studies in South Asian countries, including Bhutan, India, and Nepal, reported that the proportion of post-term births ranged from 2.1–6.58% [27–30]. Most studies used hospital-based data and did not report the population-based proportion of post-term births. The proportion of post-term births in high-income countries with high-quality data was reported to vary from 0.4–7.0% [2,4,42,43]. However, those figures were influenced by labour induction per WHO guidelines [44] for prolonged pregnancies in all clinics. Furthermore, most studies above measured the post-term birth proportions among births at or beyond 37 gestational weeks. We observed a substantial decline in post-term birth proportions starting in 2008. This calendar time corresponds to the timing of an intervention study in the area that aimed to improve antenatal and delivery care [34]. We also observed a substantial rise in full-term births commencing in 2008, which might also be influenced by the decreased proportion of pre-term births (**Figure 2**). In addition, the increase in the number of private hospitals contributing to high caesarean deliveries [45] may also lead to an increase in the proportion of full-term births, thus influencing the observed post-term birth reduction.

Our study observed that post-term births (≥42 + 0/7 weeks) were associated with increased perinatal death compared to full-term births (39 + 0/7 to 40 + 6/7 weeks), and this finding is consistent with other studies [5,16,19,46]. Furthermore, the risk of perinatal death increased even in late-term births (41 + 0/7 to 41 + 6/7 weeks) [47,48]. Foetal growth restriction or small for gestational age could occur in post-term birth that might increase the risk of perinatal death due to a poorly functioning placenta that fails to supply appropriate nutrients and oxygen to the foetus [4,22]. In addition, a connection between post-term birth and placental ageing, as well as between placental ageing and perinatal morbidity, could explain the increased risk of perinatal death [18]. Congenital malformations may also explain part of this increased risk of perinatal death [22]. However, due to a lack of clinical information, we were unable to determine the role of the above clinical features on perinatal death. In contrast, some studies observed no association between stillbirths or neonatal death and post-term births [7,42,49,50]. This lack of association may partially be explained by late antenatal booking, resulting in misclassification due to long recall periods of LMPs and an increased number of labour induction and operative deliveries, causing a smaller number of post-term births left for risk estimation.

The present study observed a substantial decline in perinatal mortality between 1990 and 2019 in rural Matlab, Bangladesh. The pace of reduction of perinatal deaths in this study is higher than reported in the recent 2017–18 Demographic and Health Survey in Bangladesh (48 per 1000 births) [24]. This study found that women with no education and primiparity observed higher risks of perinatal death. The study finding is consistent with other studies conducted in low-resource settings [51–53]. The reason might be that women with no education were more likely to belong to poor households and had limited access to health care services. Primipara women were also more likely to experience pregnancy, labour, and delivery-related complications that require emergency obstetric care [52]. icddr,b has been working to improve maternal and child health for more than four decades in the study area. The results of these efforts are reflected in several improvements in maternal and child health indicators, including the reduction of perinatal deaths compared to other areas of the country [32,34,54]. Although there is a remarkable reduction in perinatal deaths at the national level [24], reaching the proposed Sustainable Development Goals targets remains a challenge for the country. Improving the care of prolonged labour remains a window of opportunity to further improve the stillbirths and neonatal deaths in low- and lower-middle-income countries.

The current study has several strengths. The study used prospectively collected population-based information from a well-defined rural community in a low-resource setting. The population-based HDSS databases, collected by an independent group of health workers, and the inclusion of more than 90% of birth cohorts in the analysis limited the risk of information and selection biases. The large sample size allowed us to generate results with high precision and evaluate the mortality risks by stratifying by gestational weeks at births and by study periods. The covariate information retrieved from the HDSS databases maintained a high accuracy [32]. We excluded only 9% of births from the analysis due to missing information on covariates or gestational age at births, and this small proportion of exclusion was unlikely to influence the overall findings. The reported LMP date used to ascertain gestational age may raise concerns about internal validity. An earlier study, using the same data as the present study, assessed the validity of gestational age estimates based on reported LMP compared to ultrasound estimates and found Cronbach's alpha to be 0.89, demonstrating high agreement [32,55]. Furthermore, any misclassification of births by gestational age probably occurred randomly, thus attenuating the observed risk estimation in our study.

However, some weaknesses of the study should be highlighted. The Matlab HDSS started to collect data on the mode of delivery (vaginal and caesarean deliveries) in 2005. Thus, we lacked data before that year. However, when stratifying data on vaginal and caesarean deliveries, we observed similar risk estimates of perinatal mortality between 2005–19. Our population-based HDSS databases lacked clinical data of pregnant women and foetuses, delivery information including labour induction, and antenatal service utilisation data. Therefore, we could not explore the risks of perinatal mortality in prolonged pregnancies by clinical groups or prenatal service utilisation patterns.

The findings of the present study have clinical and public health importance. The study provided evidence that full-term births had the best perinatal outcomes [20,56]. Practitioners and policymakers should recognise the importance of valid LMP date recording, bringing all pregnant women who are 40 + 0/7 to 40 + 6/7 weeks of gestation, and establishing the ability of foetal surveillance in the facilities. Furthermore, the PAF of births beyond 41 + 0/7 gestational weeks implied that about one in seven perinatal deaths could be prevented if the health systems follow the guidelines to avoid prolonged pregnancy and adopt evidence-based labour induction programs for birth planning at 39 + 0/7 to 40 + 6/7 weeks of gestation. All these efforts may improve perinatal health outcomes and thus may play a role in achieving the global targets for stillbirth and neonatal death reduction in low- and lower-middle-income countries.

Further research needs to be conducted in other geographical areas in Bangladesh and other low- and lower-middle-income countries with high perinatal mortality rates to determine the occurrence of post-term births and their association with perinatal death. In addition, studies are needed to determine the biological pathways and the mechanisms behind perinatal deaths in post-term births. Furthermore, evaluations and implementation research are needed on the management of women approaching the post-term period on the reduction of perinatal death. There is a concern about increased caesarean section rates due to post-term birth. At 41 + 0/7 weeks of gestation, WHO recommends initiating labour induction. However, studies that followed the WHO guidelines on prolonged labour management reported no increase in caesarean section rate and also observed a reduction in perinatal deaths [57,58].

## CONCLUSIONS

In conclusion, this study has provided unique information on the distribution of gestational age at birth and the associated perinatal mortality of post-term births in a rural population in Bangladesh. Our findings suggest an additional risk of perinatal deaths when the pregnancy continues at ≥41 weeks. Increased and sustained efforts may need planned management of pregnant women approaching the post-term period to reduce perinatal mortality.

## Additional material


Online Supplementary Document
