# Supplementary material for: A cohort study of the occurrence of post-term births and its association with perinatal mortality in a rural area in Bangladesh
Source: J Glob Health. 2024 Nov 15;14:04238. doi: 10.7189/jogh.14.04238 (PMC11565093; doi:10.7189/jogh.14.04238)

Online Supplementary Document

Table S1. Association of covariates (maternal age, maternal education, parity, and birth year) with perinatal death in the pregnancy cohorts in Matlab, Bangladesh (n=72373)

	Unadjusted		Adjusted*	
	RR (95% CI)	*P*-value	aRR (95% CI)	*P*-value
Maternal age (years)				
<20	1.13 (1.02-1.25)	0.018	1.12 (1.01-1.24)	0.025
20-24	1.00		1.00	
25-29	0.90 (0.82-1.00)	0.050	0.90 (0.82-1.00)	0.047
≥30	1.20 (1.09-1.33)	<0.001	1.12 (1.02-1.24)	0.020
Education (years)				
0	2.25 (1.79-2.82)	<0.001	1.70 (1.36-2.14)	<0.001
1-5	1.60 (1.27-2.02)	<0.001	1.31 (1.04-1.65)	0.022
6-10	1.29 (1.02-1.62)	0.030	1.19 (0.95-1.49)	0.127
≥11	1.00		1.00	
Parity				
0	2.01 (1.84-2.19)	<0.001	2.18 (2.00-2.38)	<0.001
1	0.88 (0.79-1.00)	0.028	0.97 (0.86-1.07)	0.534
≥2	1.00		1.00	
Birth year				
1990-1999	2.22 (2.01-2.44)	<0.001	1.71 (1.54-1.89)	<0.001
2000-2009	1.70 (1.53-1.88)	<0.001	1.50 (1.36-1.67)	<0.001
2010-2019	1.00		1.00	

RR – relative risk, aRR – adjusted relative risk, CI – confidence interval.
*adjusted for gestation weeks at birth.

Table S2. Relative risk of perinatal death by each gestation week in Matlab, Bangladesh

	Unadjusted RR (95% CI)	*P*-value	Adjusted RR* (95% CI)	*P*-value
GA (weeks) at delivery				
37 vs 39 (Ref 39)	1.49 (1.27-1.75)	0.000	1.44 (1.23-1.69)	0.000
38 vs 39 (Ref 39)	0.98 (0.84-1.15)	0.842	0.98 (0.83-1.14)	0.756
40 vs 39 (Ref 39)	1.06 (0.91-1.24)	0.417	1.06 (0.92-1.24)	0.420
41 vs 39 (Ref 39)	1.50 (1.27-1.77)	0.000	1.44 (1.22-1.69)	0.000
42 vs 39 (Ref 39)	2.12 (1.73-2.61)	0.000	1.81 (1.47-2.22)	0.000
43 vs 39 (Ref 39)	2.16 (1.62-2.88)	0.000	1.93 (1.45-2.57)	0.000
44 vs 39 (Ref 39)	2.39 (1.63-3.51)	0.000	2.33 (1.60-3.41)	0.000

RR – relative risk, CI – confidence interval.
*adjusted for maternal age, education, parity, and birth year.

Table S3. Perinatal death risks stratified by three study periods by year from 1990 to 2019 in Matlab, Bangladesh

	1990-1999			2000-2009			2010-2019		
Gestation weeks at birth	Perinatal death n (%)	Crude RR (95% CI)	Adjusted RR* (95% CI)	Perinatal death n (%)	Crude RR (95% CI)	Adjusted RR* (95% CI)	Perinatal death n (%)	Crude RR (95% CI)	Adjusted RR* (95% CI)
Pre-term (<37)	588 (47.3)	2.87 (2.49-3.31)	2.79 (2.42-3.22)	365 (37.3)	3.66 (3.12-4.29)	3.59 (3.05-4.22)	250 (44.0)	6.47 (5.32-7.86)	6.29 (5.17-7.66)
Early-term (37-38)	224 (18.0)	1.07 (0.90-1.28)	1.08 (0.91-1.28)	190 (19.4)	1.22 (1.01-1.48)	1.26 (1.05-1.52)	99 (17.4)	0.99 (0.77-1.27)	1.04 (0.81-1.34)
Full-term (39-40)	257 (20.7)	1	1	236 (24.1)	1	1	158 (27.8)	1	1
Late-term (41)	93 (7.5)	1.47 (1.17-1.86)	1.46 (1.16-1.83)	92 (9.4)	1.40 (1.11-1.78)	1.35 (1.06-1.70)	42 (7.4)	1.32 (0.94-1.85)	1.31 (0.93-1.83)
Post-term (42-44)	80 (6.4)	1.64 (1.28-2.10)	1.66 (1.30-2.11)	96 (9.8)	2.19 (1.73-2.76)	2.12 (1.69-2.67)	19 (3.4)	1.68 (1.05-2.68)	1.67 (1.05-2.67)

RR – relative risk, CI – confidence interval.
*adjusted for maternal age, education, and parity.

Table S4. Risk of perinatal deaths stratified by delivery types by year from 2005 to 2019 in Matlab, Bangladesh

	Vaginal delivery			Caesarean delivery		
	N	Crude RR (95% CI)	Adjusted RR* (95% CI)	N	Crude RR (95% CI)	Adjusted RR* (95% CI)
Gestation weeks at birth						
Pre-term (<37)	3076	5.65 (4.74-6.73)	5.18 (4.34-6.18)	1328	5.06 (3.66-7.01)	4.91 (3.52-6.86)
Early-term (37-38)	6670	1.19 (0.96-1.48)	1.20 (0.97-1.48)	3528	0.94 (0.64-1.39)	0.96 (0.64-1.44)
Full-term (39-40)	11543	1.00	1.00	4561	1.00	1.00
Late-term (41)	2591	1.40 (1.05-1.85)	1.37 (1.03-1.82)	1139	1.70 (1.07-2.70)	1.69 (1.06-2.69)
Post-term (42-44)	1254	2.37 (1.75-3.21)	2.30 (1.71-3.10)	432	2.14 (1.16-3.96)	2.11 (1.15-3.90)

RR – relative risk, CI – confidence interval.
*adjusted for maternal age, maternal education, and parity.

Table S5. Population-attributable fraction (PAF) of perinatal death stratified by gestation weeks at birth by year from 1990 to 2019 in Matlab, Bangladesh

	**Birth years**											
	**1990-1999**			**2000-2009**			**2010-2019**			**All**		
	**n=11218**			**n=14082**			**n=14262**			**n=39562**		
Gestation weeks at birth	RR* (95% CI)	**P**	PAF (95% CI)	RR† (95% CI)	**P**	PAF (95% CI)	RR‡ (95% CI)	**P**	PAF (95% CI)	RR§ (95% CI)	P	PAF (95% CI)
41-44 weeks	1.53 (1.27-1.85)	30	13.7(12.1-15.3)	1.64 (1.36-1.98)	32	17.0 (15.8-18.2)	1.41 (1.05-1.90)	21	7.9 (6.3-9.56)	1.63 (1.45-1.84)	28	15.0 (14.3-15.7)
39-40 weeks	1.00	70		1.00	68		1.00	79		1.00	72	

RR – relative risk, **P** – Prevalence is provided as percent, CI – confidence interval.
*adjusted for women's age, women's education, and parity.
†adjusted for women's age, women's education, and parity.
‡adjusted for women's age, women's education, and parity.
§adjusted for women's age, women's education, and parity.

Figure S1. Distribution of pre-term (<37 weeks), early-term (37+0/7 to 38+6/7 weeks), full-term (39+0/7 to 40+6/7 weeks), late-term (41+0/7 to 41+6/7 weeks), and post-term (42+0/7 to 44+6/7 weeks) births by caesarean delivery from 2005-2019.

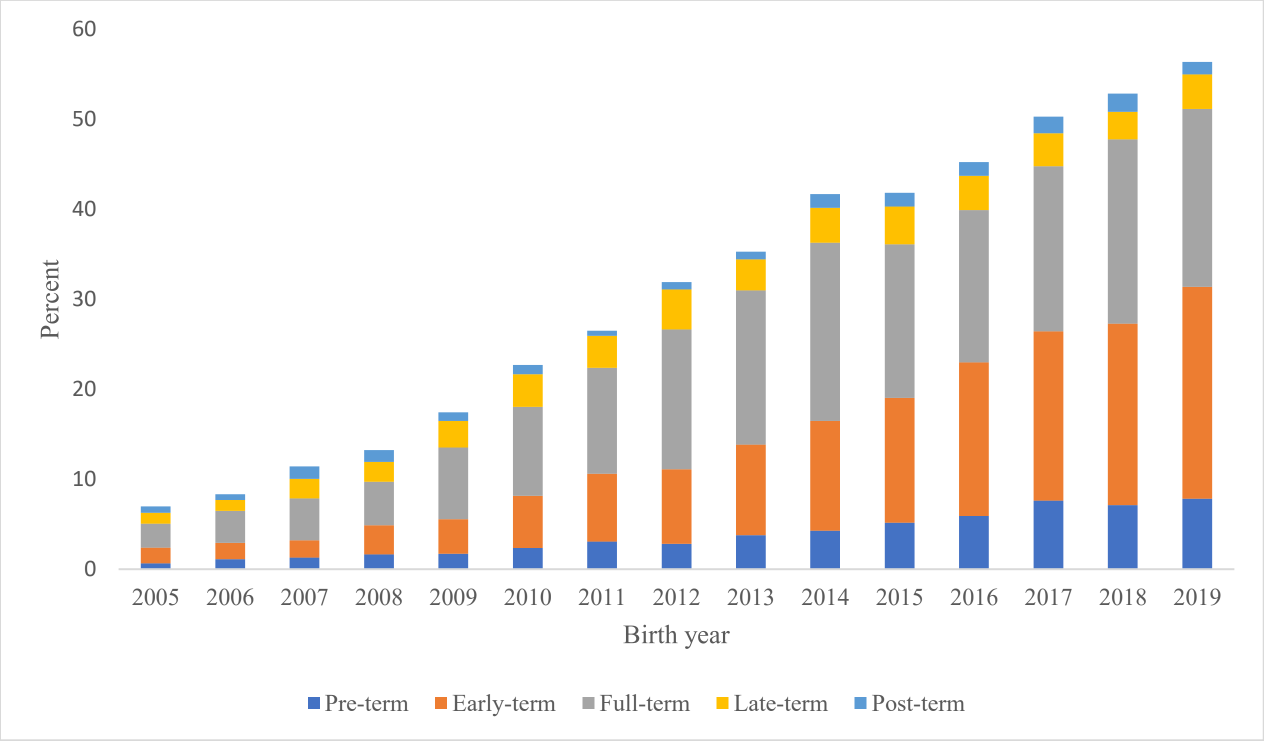

Supplement: Online Supplementary Document [file jogh-14-04238-s001.pdf]
